# Effects of Rural Medical Insurance on Chronically Ill Patients’ Choice of the Same Hospital Again in Rural Northern China

**DOI:** 10.3390/ijerph15040731

**Published:** 2018-04-12

**Authors:** Ke Jiang, Daming You, Zhendong Li, Wei Wei, Mitchell Mainstone

**Affiliations:** 1 School of Business, Central South University, Changsha 410083, Hunan, China; nicolejiang@csu.edu.cn (K.J.); youdaming2001@163.com (D.Y.); 2 College of Management and Economics, Tianjin University, Tianjin 300072, China; 3 Manchester Institute of Innovation Research, Alliance Manchester Business School, The University of Manchester, Manchester M13 9PL, UK; 4 Department of Scientific Research, Tianjin University of Traditional Chinese Medicine, Tianjin 300193, China; weiwei@mail.tjutcm.edu.cn; 5 Hertford College, University of Oxford, Oxford OX1 3BW, UK; mitchell.mainstone@hertford.ox.ac.uk

**Keywords:** patients with chronic diseases, patient’s choice of hospital, rural medical insurance, Northern China

## Abstract

The emergence of rural health insurance plays a crucial role in alleviating the pressure on rural medical expenditure. Under the current medical system in northern China, rural medical insurance may reduce the free referral of patients with chronic diseases among hospitals. This study was carried out based on the results of an investigation of rural chronically-ill patients in eight county hospitals in northern China, as well as through the comparison and analysis of patients with chronic diseases, considering whether they were with or without rural health insurance. The main results showed that both age (*χ*2 = 22.9, *p* < 0.000) and income level (*χ*2 = 18.5, *p* < 0.000) had considerable impact on the rural peoples’ willingness to buy health insurance. Meanwhile, both the quality of the hospital’s treatment (*B* = 0.555, *p* < 0.000), and service quality (*B* = 0.168, *p* < 0.000) had a significant positive correlation with the likelihood of a given patient choosing the same hospital on the next visit, but the medical costs had a significant negative correlation (*B* = −0.137, *p* < 0.000). Eventually, it was found that the provision of rural health insurance had weakened the three relationships upon which the aforementioned correlations were based.

## 1. Introduction

In recent years, with the rapid development taking place across both the economy and wider cultural spheres of China, the incidence of non-communicable chronic diseases has been rising rapidly. This is especially so in rural northern China, owing to a generally rural, younger labor force entering the urban labor market, coupled with a lowering of the birth rate over time. This has led to an increasingly aged population, which has been highlighted by the proportion of the population suffering from chronic diseases increased year on year in rural northern China [1,2]. Practically, chronic diseases take on prominent long-term feature and the treatment process tends to be lifelong. The chronically-ill patients in rural areas are mostly elderly, with their income levels being relatively low [3]. Therefore, self-financed medical treatment will exert greater financial pressure on these elderly people. Under such circumstances, the emergence of rural medical insurance plays an important role in easing the stress on their medical expenditure [4].

Currently, the provision of medical insurance in rural China is mainly funded through national government policy, with local governments and farmers forming a special health fund, which reimburses medical expenses up to a defined percentage of the total cost (generally 60%). This is classed as a voluntary (opt-in) medical cooperation system, and owing to the different local policies, the specific content and reimbursement ratio of medical insurance are also not the same. The rural medical insurance funds in counties and districts are managed by the local government. This has led to the local government restricting patients with local medical insurance to receiving treatment only in local hospitals, thereby increasing the income of local medical industry and reducing their county and/or district’s potential fiscal deficits [5,6]. Therefore, the administrative department of public health often increases institutional barriers in the process of referring patients to outside hospitals. This has caused patients who have rural medical insurance to be unable to be transferred easily to other hospitals. Especially in northern China, chronically ill patients cannot transfer between hospitals conveniently under the current medical insurance system, due to an economic gap between the northern and southern areas, imperfect medical insurance system, and external influence of the local government to the medical environment. Thus, it can be said that the rural health insurance will, to a certain extent, affect the selection of clinic hospitals for the next treatment based on the satisfaction of the patients with chronic diseases in northern China [7,8]. Therefore, by a comparative analysis on chronic patients both with and without medical insurance in rural northern China, this study aimed to investigate how rural medical insurance affects patients with chronic diseases, in terms of their choosing a particular hospital for medical treatment, and whether this will alter their repeat choice of hospital in the future.

Regarding the definition of patient satisfaction, this study refers to the concept of customer satisfaction proposed by Oliver [9], who pointed out that customer satisfaction depends on the gap between customer expectations and perceived performance of products or services. The author described customer satisfaction as a kind of rational cognition process, and emphasized the influence of cognitive processes on customer satisfaction. Later study found that emotion is another important factor influencing customer satisfaction, and even an indispensable antecedent variable in the process of consumption [10]. In patient satisfaction, this part of emotion probably originated from the perception of its quality during the service. As such, Park and Cho highlighted that customer satisfaction involves four aspects: cognitive state, cognitive assessment, emotional response and satisfaction judgment [11]. Multiple references to the application of customer satisfaction theory in patient treatment satisfaction can be found in below. In 1984, Flexner and Berkowitz [12] argued that patients determine the clinic hospitals merely through four factors: physician strength, hardware equipment, medical service quality and medical image and reputation. On this basis, Boscarino and Stelber [13] elevated the price of medical service as an important supplement, and stressed that the hospital rankings and others’ recommendation indirectly affect patients’ choice of hospital. Subsequently, Kurz and Wolinsky [14] proposed four key factors influencing factors for patients’ selection in hospitals: knowledge information, cost, medical quality and referrals. Taylor and Capella [15] further indicated that a patient’s choice of hospital depends on its appearance, reputation, and how employees interact with patients. Additionally, the overall reputation of hospital, patients’ previous medical experience, risk appetite and external medical service cost have also been supplemented by many scholars [16,17,18] as imperative factors in selecting hospital.

Accordingly, hospital’s treatment levels [19], service quality [20,21] and medical costs [22], as perceived by chronic patients themselves, all determine their satisfaction with the hospital, which in turn ultimately affect the patient’s next choice of hospital that provides their treatment. However, we have found that, in northern China, chronically-ill patients with rural medical insurance are more accustomed to be treated at the same hospital on the next visit, while far fewer in various hospitals, especially those that are located further away. In view of this, this study tried to explore how rural medical insurance in northern rural areas affects patients with chronic diseases selection of the same hospital upon the next treatment.

## 2. Materials and Methods

### 2.1. Study Design and Sampling

Our study is closely related to previous work under the title of “Study on the influencing factors for patients with chronic diseases on hospital choice in northern China”, for which research was conducted between August 2016 and November 2016 in Changsha, Taiyuan and Tianjin. Due to the disparity in the level of economic development, the medical environment in north China and south China is not completely equivalent. For example, the level of residents’ consumption, the willingness of residents to purchase medical insurance, and the subsidy of local governments will all have a certain effect on the selection of hospitals for rural patients with chronic diseases. In view of this, under the current medical environment in China, the choice of how rural medical insurance in northern rural areas affect the selection of medical treatment for rural chronic patients is more representative. The study was based on research conducted on rural chronically-ill patients of eight County Hospitals in northern China (three hospitals in Changsha, three hospitals in Tianjin and two hospitals in Taiyuan), and evidenced through carrying out of questionnaires. Rural patients with chronic diseases who were not local residents were included if they had local rural medical insurance, or lived in the surveyed areas. Because the departments of each hospital were organized in different ways, we conducted a questionnaire to investigate all relevant departments of the chronic diseases category that fit this study. Specifically, questionnaires were distributed directly to chronic disease patients with rural household registration in the relevant departments’ entrance or treatment hall. Our investigators then gave on-site guidance and detailed explanations on how patients filled out the questionnaires, and collected them from chronic patients. Eventually, as a token of thanks, a gift was given to each patient who completed the questionnaire. In total, 1347 questionnaires were distributed, and 988 correctly completed questionnaires were received, effectively giving a, current, total response rate of 73.35%.

### 2.2. Dependent Measures

We assessed patients’ plans based on the question “If re-treatment is necessary, will you choose the same hospital again?” along with three dimensions, that is, “if necessary, I will choose the outpatient service, to be hospitalized and carry out the necessary treatment in the same hospital gain” [19,23]. Respondents indicated their agreement to this future action of the three above provided, and they answered this using a seven-point Likert-type scale with “1” equating to “strongly disagree” and “7” equating to “strongly agree”. The relative reliability of these measurements (Cronbach’s α) was 0.845.

### 2.3. Independent Measures

In China, the rating of hospitals is divided into ten levels. For the purposes of this study, we set these hospital ratings on a five-point scale, ranging from one (grade 1–2) to five points (grade 9–10). We also measure the level of medical equipment and the ability of hospital medical staff. A total, scaled score was calculated using these three items (Cronbach’s α = 0.802) [24,25].

We also included the detail questions for the measurement of services quality, such as the conveniences, service level, medical ethics and trust [20,26,27]. The responses were measured on the aforementioned seven-point scale from “1” (strongly disagree) to “7” (strongly agree). This scale yielded a Cronbach’s α of 0.823.

To measure the perceived medical costs for chronically-ill patients, we analyzed patients’ answers to posed questions, such as the costs of outpatient service, medicine and nursing service [22,28,29]. These three key components of patients’ medical expenses were scored on the same seven-point scale. The relative reliability of these measurements (Cronbach’s α) was 0.811.

### 2.4. Statistical Analysis

The data we collected were then subject to descriptive analysis, by means of IBM SPSS Statistics 19.0 (IBM, Armonk, NY, USA). An Independent-Samples *t* test was adopted to comparing the respective means of these two measured groups. A linear regression model was employed to analyze the associations between the possibility of patients choosing the same hospital again and the potential influencing factors in their decision-making process (including age, gender, income class, and education level, as well as the hospital’s subjective treatment quality, overall service quality, and medical costs) in the group of patients without medical insurance (Group A) and that in the group of patients with medical insurance (Group B). Our aim was to illustrate how the provision of rural medical insurance in northern China affected the decision-making process of chronically-ill patients repeatedly visiting the same hospitals, for the treatment necessitated by their respective conditions. Accordingly, we first divided the rural chronically-ill patients into two groups according to whether they did or did not possess rural medical insurance. Afterwards, the significant differences between the two groups in regards to influencing factors affecting them, including age, gender, educational background and income, were calculated. Subsequently, within both groups (including their respective influencing factors), the potential correlations between “the possibility of patients choosing the same hospital on next visit” and their current hospital’s “Quality of treatment”, “Overall service quality”, and “Medical cost” was analyzed. Eventually, the changes in the correlation coefficients between the two groups were compared.

Adjustments were made according to the distribution of patient age [30], gender, income class [1,31], and education level and disease category. The dummy variables were established to measure gender (where Female is equated to “0” and Male is equated to “1”) as well as disease categories (equating random diseases with the value of “1”, and others with “0”), while age and income classes were adopted to measure the aforementioned five-point Likert-scale, ranging from score “1”, equated to the values “Age: <30 years/Income: <RMB 2500” to score “5”, which was equated to the values “Age: >75 years/Income: >RMB 12,000”. A two-tailed significance level of *p*-value < 0.05 was consequently used.

### 2.5. Ethics Statement

This study protocol was approved by the Tianjin University of Traditional Chinese Medicine (TJUTCM) Ethics Committee. The reference number is TJUTCM-EC20160005.

## 3. Results

### 3.1. Sample Characteristics

**Figure 1:**
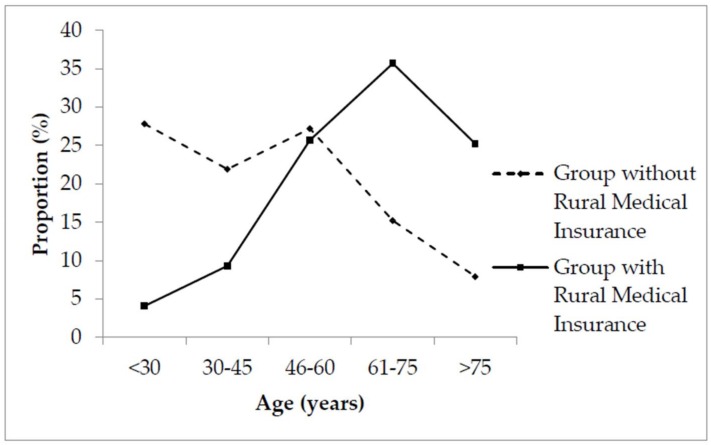
The proportions of patients with chronic diseases of all ages.

**Figure 2:**
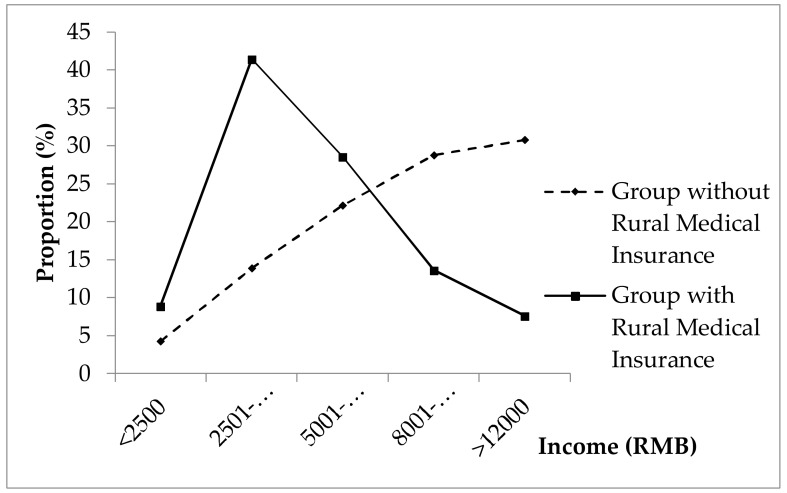
The proportions of patients with chronic diseases of income classes.

A total of 988 participants were involved in this study, including 452 females (45.7%) and 536 males (54.3%). The average age of respondents was 52 years old (±3.0 years). There were 686 (69.43%) participants belonging to the group of patients with rural medical insurance and 302 (30.57%) participants belonging to the group without rural medical insurance. The characteristics of the demographic of the sample taken and their significant differences are shown in Table 1 (the total percentage of disease category are larger than 1 because some patients had more than chronic diseases). These differences were found across both age (χ^2^ = 22.90, *p* < 0.000) and income class (χ^2^ = 18.50, *p* < 0.000). Among them, especially compared with the group having medical insurance, the age composition of patients without medical insurance tended to be younger (see in Figure 1), while the income level of the group without medical insurance was higher (see in Figure 2). Additionally, there were no significant differences observed across gender (χ^2^ = 0.85, *p* = 0.106), education (χ^2^ = 1.68, *p* = 0.683), and disease category proportions (as shown in Table 1).

### 3.2. Association between the Possibility of Patients Choosing the Same Hospital Again, and Treatment Quality, Medical Services Quality and Medical Costs

The respective correlation coefficients between a given patient’s likelihood of choosing the same hospital upon their next visit and either their perception of the quality of their treatment, the service quality, or the medical costs incurred are shown in Table 2. In the sample of patients surveyed, the perceived quality of treatment provided by the hospital was positively correlated to the patient’s choice of the same hospital upon their next necessary visit (*B* = 0.555, *p* < 0.000). Similarly, a hospital’s entire service quality also had a significant positive impact as to whether the patient would continue to choose the same hospital again (*B* = 0.168, *p* < 0.000). Contrarily, there was a significant negative relationship between medical costs incurred and the possibility of the patient choosing the same hospital on next visit (*B* = −0.137, *p* < 0.000).

Besides these features, we also noted that age was positively related to the possibility of that patient choosing the same hospital again (*B* = 0.072, *p* = 0.007). There was also a significant positive correlation between income level and the possibility of opting for the continuance of treatment at the same hospital upon a patient’s next necessary visit (*B* = −0.072, *p* = 0.008). However, interestingly, there was no significant correlation between level of patient education and the probability of choosing the same hospital again (*B* = −0.005, *p* = 0.851).

### 3.3. The Role of Rural Medical Insurance on the Patient’s Choice of Hospital upon Next Visit

In analyzing the behaviors of rural patients without medical insurance (Group A), and those with medical insurance (Group B), as shown in Table 2, we found that the respective correlation coefficients between a hospital’s perceived treatment quality, overall service quality, and medical expenses incurred and the possibility of patient choosing the same hospital again in two groups all varied significantly (except the variable of medical cost in Group B). As shown in Table 2 and Table 3, the provision of rural medical insurance, with the given data, significantly weakened the positive relationship between perceived quality of treatment, and the likelihood of choosing the same hospital again (correlation coefficient *B* of Group A (*B*A) = 0.698, correlation coefficient *B* of Group B (*B*B) = 0.441, *p* < 0.004), as well as the positive relationship between entire service quality perceived by the patient in the hospital and the possibility of choosing the same hospital again (*B*A = 0.239, *B*B = 0.102, *p* < 0.000). In addition to these points, the provision of rural medical insurance itself also weakened the negative relationship between the medical expense of patient and the possibility of choosing the same hospital again upon the patient’s next visit (*B*A = −0.225 *B*B = −0.045, *p* = 0.003). Furthermore, there were no significant differences in the correlations of age (*B*A = 0.064, *p* = 0.129, *B*B = −0.010, *p* = 0.776), gender (*B*A = 0.034, *p* = 0.413, *B*B = 0.044, *p* = 0.195), income level (*B*A = −0.061, *p* = 0.141, *B*B = −0.004, *p* = 0.914) or educational background (*B*A = 0.035, *p* = 0.398, *B*B = −0.023, *p* = 0.505) for the possibility of choosing the same hospital again within both of the two groups. In addition, the patients with rural medical insurance more likely to choose the same hospital next visit than those without rural medical insurance (mean value of Group A (*M*A) = 4.116, mean value of Group B (*M*B) = 4.493, *p* < 0.000).

## 4. Discussion

By comparing and analyzing the basic information provided about patients, we found that there was a significant difference in age between the groups with and without rural medical insurance. To be precise, the age level of patients without rural medical insurance was younger than the alternate group who were insured. This could be due to large proportions of young migrant workers unable to enjoy the convenience of local medical insurance. Besides, other factors affecting these results may include young people, whom perhaps believing that the probability of their becoming ill is extremely low, thus reducing their willingness to apply for medical insurance [32,33]. It was noteworthy that income class was a significant factor between the two groups, to the extent that the income level of the uninsured group was significantly higher than that of the insured group. The reason for this difference may be due to the fact that the willingness of prosperous rural entrepreneurs and wealthier farmers to participate in rural medical insurance is not particularly strong, regarding their wealth. On the contrary, the lower income earners demonstrated a stronger desire to take out rural medical insurance [34,35].

The sample was divided into two groups: chronically-ill patients with rural medical insurance, and those without rural medical insurance. This then allowed us to use regression analysis to learn that rural medical insurance reduced the positive relationship between the perceived quality of the hospital’s treatment and the patient’s continued choice of the hospital on subsequent visits. Meanwhile, the positive correlation between the quality of the services provided in the hospital, and the patients’ continued choice of the hospital for future treatment also weakened, as did the negative correlation between the medical expenses of patient and the patients’ continued choice of the hospital for their future care. This could be because, in China, the rural medical insurance budget is usually coordinated with counties and districts, each area has a corresponding amount of health insurance funds. In this case, if patients were allowed to transfer freely to hospitals in different areas, the outflow of medical insurance funds of the local government and the income levels of local hospitals will be rapidly depleted simultaneously. Beside, chronically diseases diagnosis and treatment program of most of hospital are similar, resulting in a more stringent approval for the referral of patients with chronic disease by the medical insurance referral department. These have made it impossible for chronically-ill patients, particularly those with rural health insurance, to freely change the hospital at which they are treated. In other words, even if patients with chronic diseases show lower evaluation on perceived treatment levels, quality of service and expense of the hospital, as a result of poor rural medical insurance mobility, patients may also continue to tolerate and accept treatment in the same hospitals where their needs are continually going unmet. While chronically-ill rural patients without medical insurance can freely choose another (potentially better) hospital for medical treatment, based on perceived quality of treatment, service and price level of the hospital, they can do this entirely due to the self-financed status of their medical treatment to begin with [36]. Therefore, rural medical insurance reduces the possibility of rural patients with chronic diseases choosing a hospital according to optimal levels of these aforementioned factors, and consequently restricting the choices available to them.

## 5. Conclusions

By a comparative analysis on chronic patients both with and without medical insurance in rural northern China, this study aimed to investigate how rural medical insurance affects patients with chronic diseases, in terms of their choosing a particular hospital for medical treatment, as well as the possibility of these patients opting to choose the same hospital again in the future. Based on the results of an investigation of rural chronically-ill patients in eight county hospitals in northern China, we found that both age and income level have considerable impact on the rural peoples’ willingness to buy health insurance. Meanwhile, both the quality of the hospital’s treatment, and service quality had a significant positive correlation with the likelihood of a given patient choosing the same hospital on the next visit, but the medical costs had a significant negative correlation. Eventually, we discovered that the provision of rural medical insurance can significantly weaken the correlativity between the above three factors and the patient’s choice of same hospital upon their next visit.

This study presents several limitations. First, because the level of economic development and the medical environment in north China and south China are not completely equivalent, this study merely focuses on the questionnaire survey in northern China, so the universality of the conclusion needs further verification. Moreover, during the questionnaire distribution, regional differences or subjective causes may directly affect our research results. The study did not consider more factors that may affect the patient’s purchase of medical insurance, such as “risk appetite.” As such, how to improve the universality of research conclusions by expanding the sample size and rationalizing sample distribution will be considered in further studies.

## Figures and Tables

**Table 1:** Participants’ Demographic Characteristics.

Item	No Insurance (A)		Insurance (B)		χ^2^	*p*
	*N*	*%*	*N*	*%*		
Age:					22.90	<0.000
<30	84	27.8	28	4.1		
30–45	66	21.9	64	9.3		
46–60	82	27.2	176	25.7		
61–75	46	15.2	245	35.7		
>75	24	7.9	173	25.2		
Gender:					0.85	0.106
Female	135	44.7	317	46.2		
Male	167	55.3	369	53.8		
Education level:					1.68	0.683
Primary School	30	9.9	70	10.2		
Junior School	129	42.7	277	40.4		
Senior School	83	27.5	193	28.1		
Junior College	32	10.6	90	13.1		
Higher College	28	9.3	56	8.2		
Income Level (RMB):					18.50	<0.000
<2500	13	4.3	61	8.9		
2501–5000	42	13.9	284	41.4		
5001–8000	67	22.2	196	28.6		
8001–12,000	87	28.8	93	13.6		
>12,000	93	30.8	52	7.6		
Disease category:						
Hypertension	25	8.3	40	5.8	8.06	0.15
Coronary heart disease	35	11.6	77	11.2	0.11	0.87
Chronic kidney disease	38	12.6	85	12.4	0.03	0.93
Diabetes	36	11.9	119	17.3	16.02	0.11
Chronic liver disease	37	12.3	93	13.6	0.30	0.78
Chronic respiratory disease	42	13.9	89	13.0	0.63	0.69
Other chronic diseases	92	30.5	194	28.3	1.86	0.49

**Table 2:** Comparison of correlations between relevant, individual influencing factors and the patients’ choice of the same hospital again.

Variable	No Insurance (A)				Insurance (B)				Total (T)			
	*Β*	*p*	*Β*	*p*	*Β*	*p*	*Β*	*p*	*Β*	*p*	*Β*	*p*
Control variable:												
Age	0.029	0.613	0.064	0.129	0.023	0.548	−0.010	0.776	0.068	0.034	0.072	0.007
Gender	−0.013	0.823	0.034	0.413	0.031	0.418	0.044	0.195	0.012	0.703	0.036	0.178
Income	−0.081	0.163	−0.061	0.141	−0.015	0.693	−0.004	0.914	−0.074	0.022	−0.072	0.008
Education Level	0.070	0.230	0.035	0.398	−0.019	0.626	−0.023	0.505	0.018	0.580	0.005	0.851
Independent variable:												
Treatment Level			0.698	0.000			0.441	0.000			0.555	0.000
Services Quality			0.239	0.000			0.102	0.014			0.168	0.000
Medical Costs			−0.225	0.000			−0.045	0.317			−0.137	0.000

Dependent Variable: the patients’ choice of the same hospital upon next visit.

**Table 3:** A simple mean test between two sample groups.

Variable	No Insurance (A)		Insurance (B)		T Text		
	M	*S* *.D.*	M	*S.D.*	F	T	*p*
Treatment Level	4.275	1.285	4.045	0.996	111.53	−2.849	0.004
Services Quality	3.675	0.921	3.252	1.445	225.10	−5.152	0.006
Medical costs	3.050	1.344	3.298	1.103	53.06	2.963	0.003
Hospital choice for next visit	4.116	1.419	4.493	1.204	19.538	4.177	0.000

## References

[1] Zhang Y., Ou F., Gao S., Gao Q., Hu L., Liu Y. (2015). Effect of low income on health-related quality of life: A cross-sectional study in Northeast China. Asia Pac. J. Public Health.

[2] Su Y., Ma Y., Rao W., Yang G., Wang S., Fu Y., Liu Y., Zhang Y., You Y., Yu Y. (2016). Association Between Body Mass Index and Diabetes in Northeastern China Based on Dose-Response Analyses Using Restricted Cubic Spline Functions. Asia Pac. J. Public Health.

[3] Wang S., Kou C., Liu Y., Li B., Tao Y., D’Arcy C., Shi J., Wu Y., Liu J., Zhu Y. (2015). Rural–urban differences in the prevalence of chronic disease in northeast China. Asia Pac. J. Public Health.

[4] Jing S., Yin A., Shi L., Liu J. (2013). Whether New Cooperative Medical Schemes reduce the economic burden of chronic disease in rural China. PLoS ONE.

[5] Pan X.F., Xu J., Meng Q. (2016). Integrating social health insurance systems in China. Lancet.

[6] Liang Y., Lu P. (2014). Medical insurance policy organized by Chinese government and the health inequity of the elderly: Longitudinal comparison based on effect of New Cooperative Medical Scheme on health of rural elderly in 22 provinces and cities. Int. J. Equity Health.

[7] Yu B., Meng Q., Collins C., Tolhurst R., Tang S., Yan F., Bogg L., Liu X. (2010). How does the New Cooperative Medical Scheme influence health service utilization? A study in two provinces in rural China. BMC Health Serv. Res..

[8] Chen Y., Jin G.Z. (2012). Does health insurance coverage lead to better health and educational outcomes? Evidence from rural China. J. Health Econ..

[9] Oliver R.L. (1980). A cognitive model of the antecedents and consequences of satisfaction decisions. J. Mark. Res..

[10] Mano H., Oliver R.L. (1993). Assessing the dimensionality and structure of the consumption experience: Evaluation, feeling, and satisfaction. J. Consum. Res..

[11] Park M.H., Cho H.C. (2000). Reconceptualization and scale development of customer satisfaction. Kr. Mark. Rev..

[12] Flexner W.A., Berkowitz E.N. (1981). Media and message strategies: Consumer input for hospital advertising. Health Care Manag. Rev..

[13] Boscarino J., Stelber S.R. (1982). Hospital shopping and consumer choice. J. Health Care Mark..

[14] Kurz R.S., Wolinsky F.D. (1984). Who picks the hospital: Practitioner or patient?. Hosp. Health Serv. Adm..

[15] Taylor S.L., Capella L.M. (1996). Hospital outshopping: Determinant attributes and hospital choice. Health Care Manag. Rev..

[16] Phibbs C.S., Mark D.H., Luft H.S., Peltzman-Rennie D.J., Garnick D.W., Lichtenberg E., McPhee S.J. (1993). Choice of hospital for delivery: A comparison of high-risk and low-risk women. Health Serv. Res..

[17] Gooding S.K.S. (1996). The relative importance of information sources in consumers’ choice of hospitals. J. Ambul. Care Mark..

[18] Sloane G., Tidwell P., Horsfield M. (1999). Identification of the decision maker for a patient’s hospital choice: Who decides which hospital?. J. Hosp. Mark..

[19] Kim C.E., Shin J.S., Lee J., Lee Y.J., Kim M.R., Choi A., Park K.B., Lee H.J., Ha I.H. (2017). Quality of medical service, patient satisfaction and loyalty with a focus on interpersonal-based medical service encounters and treatment effectiveness: A cross-sectional multicenter study of complementary and alternative medicine (CAM) hospitals. BMC Complement. Altern. Med..

[20] Andaleeb S.S. (2000). Public and private hospitals in Bangladesh: Service quality and predictors of hospital choice. Health Policy Plan..

[21] Schaal T., Schoenfelder T., Klewer J., Kugler J. (2016). Determinants of patient satisfaction and their willingness to return after primary total hip replacement: A cross-sectional study. BMC Musculoskelet. Disord..

[22] Kondasani R.K., Panda R.K. (2015). Customer perceived service quality, satisfaction and loyalty in Indian private healthcare. Int. J. Health Care Qual. Assur..

[23] Lei P., Jolibert A. (2012). A three-model comparison of the relationship between quality, satisfaction and loyalty: An empirical study of the Chinese healthcare system. BMC Health Serv. Res..

[24] You L.M., Aiken L.H., Sloane D.M., Liu K., He G.P., Hu Y., Jiang X.L., Li X.H., Li X.M., Liu H.P. (2013). Hospital nursing, care quality, and patient satisfaction: Cross-sectional surveys of nurses and patients in hospitals in China and Europe. Int. J. Nurs. Stud..

[25] Schuldt J., Doktor A., Lichters M., Vogt B., Robra B.P. (2017). Insurees’ preferences in hospital choice-A population-based study. Health Policy.

[26] Deitrick L.M., Capuano T.A., Paxton S.S., Stern G., Dunleavy J., Miller W.L. (2007). Becoming a leader in patient satisfaction: Changing the culture of care in an academic community hospital. Health Mark. Q..

[27] Zhou W.J., Wan Q.Q., Liu C.Y., Feng X.L., Shang S.M. (2017). Determinants of patient loyalty to healthcare providers: An integrative review. Int. J. Qual. Health Care.

[28] Liu X., Liu Y., Chen N. (2000). The Chinese experience of hospital price regulation. Health Policy Plan..

[29] Zhao J., Zhong H. (2015). Medical expenditure in urban China: A quantile regression analysis. Int. J. Health Econ. Manag..

[30] Hopman W.M., Harrison M.B., Coo H., Friedberg E., Buchanan M., VanDenKerkhof E.G. (2009). Associations between chronic disease, age and physical and mental health status. Chronic. Dis. Can..

[31] Abegunde D.O., Mathers C.D., Adam T., Ortegon M., Strong K. (2007). The burden and costs of chronic diseases in low-income and middle-income countries. Lancet.

[32] Sun X., Jackson S., Carmichael G., Sleigh A.C. (2009). Catastrophic medical payment and financial protection in rural China: Evidence from the New Cooperative Medical Scheme in Shandong Province. Health Econ..

[33] Wang H., Zhang L., Yip W., Hsiao W. (2006). Adverse selection in a voluntary Rural Mutual Health Care health insurance scheme in China. Soc. Sci. Med..

[34] Zhang L., Wang H., Wang L., Hsiao W. (2006). Social capital and farmer’s willingness-to-join a newly established community-based health insurance in rural China. Health Policy.

[35] Yuan B., Jian W., He L., Wang B., Balabanova D. (2017). The role of health system governance in strengthening the rural health insurance system in China. Int. J. Equity Health.

[36] Barnes A.J., Hanoch Y., Rice T. (2015). Determinants of Coverage Decisions in Health Insurance Marketplaces: Consumers’ Decision-Making Abilities and the Amount of Information in Their Choice Environment. Health Serv. Res..

